# Biosynthesis of iron oxide magnetic nanoparticles using clinically isolated *Pseudomonas aeruginosa*

**DOI:** 10.1038/s41598-021-99814-8

**Published:** 2021-10-15

**Authors:** Abid Ali Khan, Sana Khan, Suhaib Khan, Simone Rentschler, Stefan Laufer, Hans-Peter Deigner

**Affiliations:** 1 Institute of Precision Medicine, Furtwangen University, Jakob-Kienzle-Straße 17, 78054 VS-Schwenningen, Germany; 2 Department of Biosciences, COMSATS University Islamabad, Park Road Tarlai Kalan, Islamabad, 45550 Pakistan; 3 Department of Pharmaceutical and Medicinal Chemistry, Institute of Pharmaceutical Sciences, Eberhard Karls University Tuebingen, Auf der Morgenstelle 8, 72076 Tübingen, Germany; 4 EXIM Department, Fraunhofer Institute IZI (Leipzig), Schillingallee 68, 18057 Rostock, Germany; 5 Faculty of Science, Eberhard Karls University Tuebingen, Auf der Morgenstelle 8, 72076 Tübingen, Germany

**Keywords:** Nanobiotechnology, Nanoparticles

## Abstract

Magnetotactic bacteria are microscale complex natural systems that synthesize magnetic nanoparticles through biologically controlled mineralization. Nanoparticles produced by this process are biocompatible due to the presence of surrounding membranes. The mechanism controlling synthesis is cost-effective and is executed by complex genomes (operons). The results are monodispersed magnetic nanoparticles displaying advantages over polydispersed ones synthesized by physical and chemical methods. In this work, we isolated *Pseudomonas aeruginosa* from clinical samples and demonstrated its ability to biosynthesize magnetic nanoparticles. *P. aeruginosa* was thrived in a carbon-minimal medium supplemented with iron at low pH. The cells aligned parallel to a magnetic field, confirming their magnetic properties. The magnetic nanoparticles were extracted, purified, and characterized using electron microscopy, magnetometry, dynamic light scattering, and X-ray diffraction. This work represents the first isolation of a magnetotactic bacterium from clinical samples. The aerobic nature of these bacteria allows them to be easily cultured under laboratory conditions, unlike their well-known microaerophilic counterparts. The biosynthesized magnetic nanoparticles can be used in many applications, including magnetic resonance imaging, diagnostics, and therapeutics (i.e., magnetic hyperthermia).

## Introduction

Magnetotactic bacteria (MTB) are bacteria whose locomotion is influenced by magnetic fields (either an applied magnetic field or the earth’s geomagnetic field)^1^. Salvatore Bellini first reported MTB in 1963. He called them magneto-sensitive bacteria^2^. Later, in 1974, Richard P. Blakemore rediscovered MTB independently and coined their current name. Blakemore explained the orientation and migration of MTB along magnetic field lines. These microbes were discovered on their response to magnetic fields, called magnetotaxis, microbial cells passively align and swim along magnetic field lines that cause their deposition at the edge of water drops in a magnetic field where they can viewed under a microscope^3^. Magnetotactic bacteria (MTB) represent a diverse group of gram negative motile, aquatic microorganism^4^. MTB have the ability to biomineralize intracellular, nano-sized inorganic magnetic crystals, called magnetosomes, through a controlled biomineralization process^5^. MTB are known to synthesize two types of minerals, iron oxides and iron sulfides. The bacteria which synthesize iron oxide biomineralize only magnetite (Fe_3_O_4_), iron sulfide producers biomineralize only greigite (Fe_3_S_4_), however, some bacteria cam yield a combination of both magnetite (Fe_3_O_4_) and greigite (Fe_3_S_4_). Biomineralized crystals are either magnetite (Fe_3_O_4_) or greigite (Fe_3_S_4_), surrounded by phospholipid bilayers^6^. Magnetite and greigite differ morphologically, but they share a single magnetic domain size range (35–120 nm). Magnetotactic behavior of MTB simplifies the search for food from three dimensions to one dimension. It is reported that magnetotaxis, in conjunction with chemotaxis, aids the MTB to locate optimal positions in vertical chemical and redox gradients for survival and reproduction. Magnetosomes organized as chains within cell, maximize the magnetic dipole moment of the cell and cause the cell to passively align along geomagnetic field^7^. Magnetic nanoparticles (MNPs) produced by these bacteria have the potential for diverse applications in health sciences and applied biology^8^. These include imaging of interior organs, precision transport of medications to their sites of action, and killing tumors through magnetic hyperthermia. Magnetosomes conjugated with proteins, glycoproteins, functional proteins/enzymes, and other compounds have biomedical properties that may be used in various applications in genetic research^9^. MTB are widespread and can be isolated from ponds, soil, oceans, and sunken mud^5^. Initially, it was thought that most MTB reside in microaerophilic environments. Therefore, substantial efforts were made to isolate them from these environments. Subsequently, MTB were isolated from aerobic environments, the current focus of most research efforts^4,10^. In aerobic environments, MTBs are easier to culture, maintain, and manipulate under lab conditions. These findings encouraged us to isolate phylogenetically-related MTB from aerobic environments.

We used *Pseudomonas aeruginosa* (a γ-proteo-bacterium) to conduct our study because iron is one of the most significant elements used by this bacterium. The importance of iron is highlighted by the fact that iron-dependent genes account for about 6% of total gene expression. Biofilm formation and intracellular signaling in *P. aeruginosa* alter iron concentrations. These changes demonstrate the significance of iron to this opportunistic pathogen^11^. The synthesis of magnetosomes is regulated by the magnetosomes gene cluster (MGC), previously known as magnetosomes island or MAI. The MGC contains genes that control magnetosome biosynthesis, determine their morphology and chemical composition. The unique MGCs are only associated with MTB. The genes essential to the biomineralization process are called mam (magnetosomes membrane) genes. Nine of them (mamA, -B, -K, -P, -Q, -E, -O, and -I) are present in all MGCs. The magnetosome genomic islands might be transmitted to other different bacteria through horizontal gene transfer (HGT)^12^.

Iron acquisition by *P. aeruginosa* occurs in the form of heme or non-heme sources. The bacteria take up iron from heme sources and utilize it through the *Phu* and *Has* systems^13^. The *Phu* system relies on membrane-bound receptors to take up heme or heme-proteins. These proteins are directly bound to receptors. The *Has* system secretes *hasAp* proteins that bind heme and make it available for the cell to be taken up via receptors (*hasR)*^14^. To use non-heme iron sources, *P. aeruginosa* produces iron-chelating siderophores that exhibit high affinity for iron. These siderophores are secreted into the local environment to chelate free iron. Two siderophores have been identified, pyoverdine and pyochelin^15^. In the present study, we report the synthesis of magnetic nanoparticles from clinical samples of *P. aeruginosa.* Previous works have used very enriched types of media full of minerals and vitamins. We used a simple chemical media containing iron salts. To the best of our knowledge, this is the first time that clinical samples of *P. aeruginosa* have been used to obtain magnetosomes through culture in a simple chemical medium.

## Results

### Colony morphology of Pseudomonas isolates and starter culture

**Figure 1:**
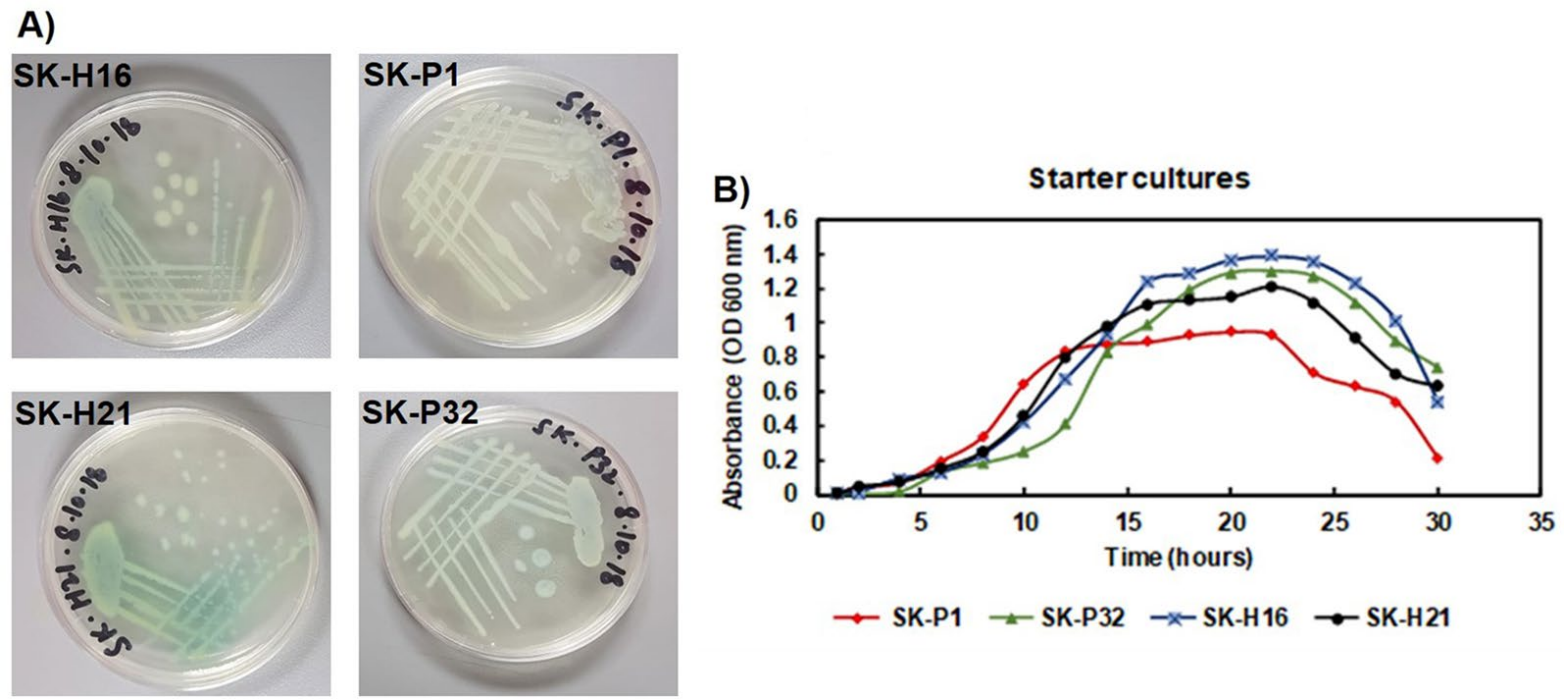
(**A**) Colony morphology of *P. aeruginosa* strains and (**B**) Growth plot SKP1, SKP32, SKH16, and SKH21 isolates in LB medium. All four strains showed the same pattern of thriving in LB medium, showing similar lag, log, stationary, and decline phases.

*Pseudomonas* colonies on nutrient agar plates of all clinical samples (SKP1, SK-P32, SKH16, and SKH21) were mucoid in appearance with greenish fluorescence (pyoverdine) (Fig. 1A) and had a grape-like odor^16^. All samples were cultured aerobically in LB medium and no fungal contamination was found after overnight incubation at 37 °C. Growth was monitored every 2 h by measuring the OD _600 nm_ of the culture. All clinical samples thrived well (Fig. 1B), and the log phase for all bacteria lasted approximately 4–5 h. The exponential phase continued for about 10 h, followed by the stationary phase. SK-H16 showed the highest cell density (OD = 1.4), while SK-P1 showed the lowest cell density (OD = 0.8). At optimal growth conditions in the log phase, glycerol stocks were prepared and stored.

### Biochemical characterization

Biochemical tests conducted reconfirm that the samples were *P. aeruginosa*. The summary of the tests is given in Table 1.

**Table 1:** Biochemical fingerprinting (tests) of *P. aeruginosa* isolates.

Sample	Oxidase	Catalase	Indole	Simmon’s Citrate	Motility	H_2_O_2_ Production
SK-H16	+	+	−	+	+	−
SK-H21	+	+	−	+	+	−
SK-P32	+	+	−	+	+	−
SK-P1	+	+	−	+	+	−

### The growth curve in 9 K medium

**Figure 2:**
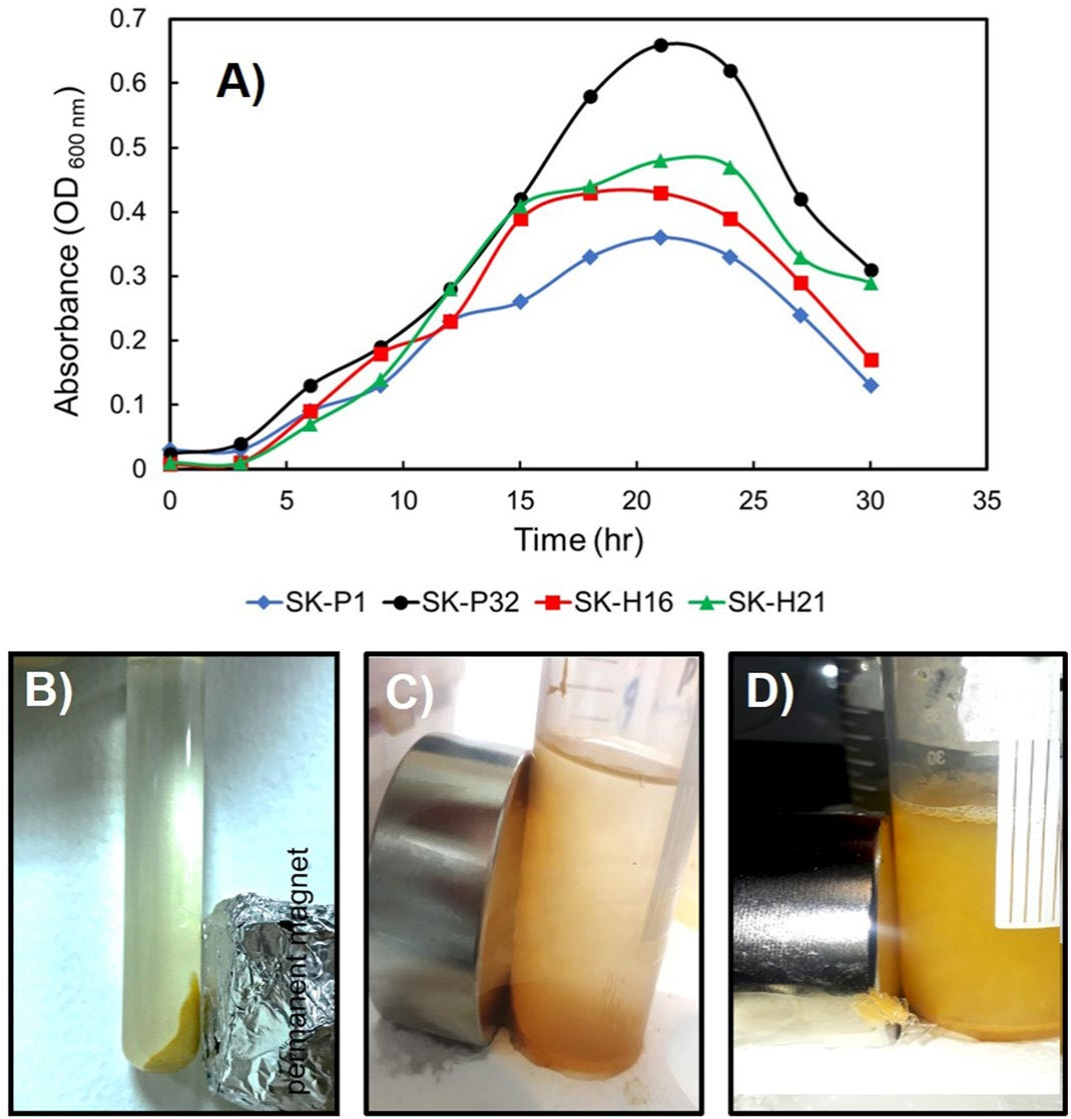
(**A**) Culture of the *P. aeruginosa* strains in 9 K medium. The longer lag and log phases show that the bacteria needed more time to adjust to the simple chemical medium. The overall ability to thrive in 9 K indicates the magnetotactic behaviour of the isolates. (**B**) The bacterial cells migrated to the side of the tube where a strong magnetic gradient was applied. (**C**) Supernatant of P. aeruginosa culture or (**D**) culture lysate did not show any movement inside the medium towards the magnetic gradient indicating that the magnetic nanoparticles are biosynthesized inside the cells only.

All four isolates were cultured in 9 K medium under low pH conditions with continuous shaking at 37 °C. The lag phase was different for each strain, ranging from 3 to 6 h (Fig. 2A). The bacteria gradually adjusted to 9 K medium. The log phase was prolonged, and most of the time was occupied with cell division. The optimal growth was in the range of 0.45 to 0.55 OD (λ = 600 nm) and continued for 30–42 h after culture initiation. The stationary phase lasted only a few hours, followed by a decline that started after 45 h. The growth plot demonstrated that the *Pseudomonas* isolates could thrive on the 9 K medium. This was a hint that the bacteria might be equipped with the capacity to biosynthesize intracellular magnetosomes/magnetic nanoparticles.

When a permanent magnet was placed to (the side of) a tube containing the bacterial cells, they aligned themselves parallel to the magnetic field gradient and stay affixed as long as the magnet was kept there (Fig. 2B). This proved that the cells were magnetic in nature. To discriminate between the possibilities whether the nanoparticles are synthesized on the cell surface or by enzymes released into the medium we cultured the cells in LB medium and used the supernatants of the culture and cell-lysate to mix with the 9 K medium. When same incubation conditions were provided, these two approaches did not result in the movement of any particles towards the strong magnetic gradient applied through an external permanent magnet (Fig. 2C–D).

### Characterization of nanoparticles

#### Imaging via electron microscopy

**Figure 3:**
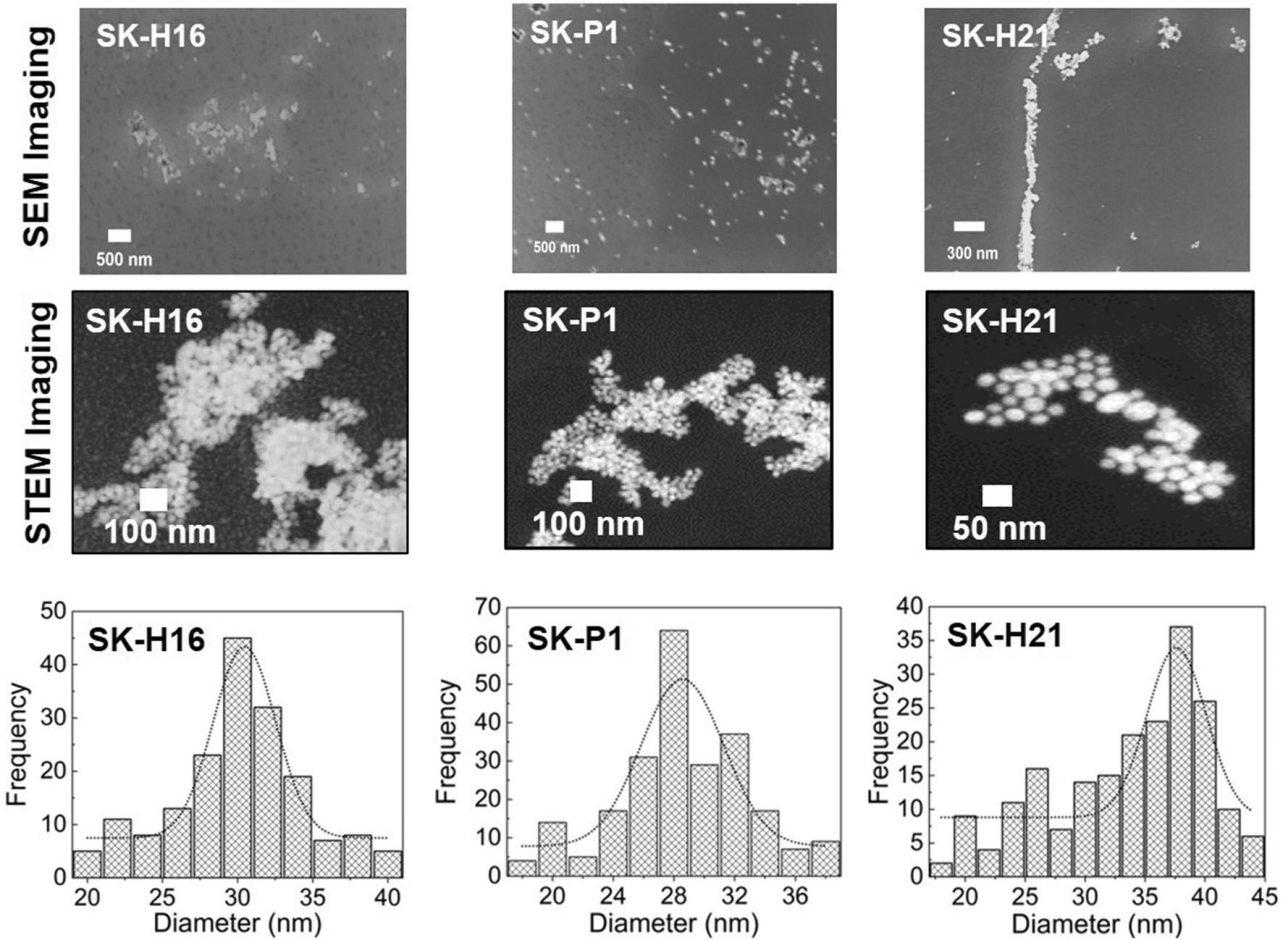
Electron microscopy images for (upper) SEM images of the magnetosome obtained from the isolates. The white spherical structures represent the magnetic nanoparticles, while the dark material represents organic matter; (middle) STEM images revealed that the magnetosomes are roughly spherical in shape for three isolates. The last row shows the magnetic nanoparticles diameter (size) distribution obtained from the STEM images.

All the characterization analysis were performed on the magnetosomes (or commonly called magnetic nanoparticles in this article) as mentioned in the materials and methods. SEM images of all four samples (Fig. 3: upper row) revealed the bright metallic architectures on the nanoscale. One of the isolates, SK-P32, however, was full of organic debris and very difficult to deal with; therefore, we considered it for further imaging characterization. However, since the images obtained with SEM were not very conclusive, we subjected the purified magnetosomes to imaging under a Scanning Transmission Electron Microscope (STEM). It can be seen that the particles were (roughly) 35–45 nm in diameter. A particle size distribution analysis obtained from the STEM images showed the diameters of all three samples (SK-H16, SK-P1 and SK-H21) were roughly the same with H21 having more particles with relatively larger diameter.

#### Dynamic light scattering (DLS)

**Figure 4:**
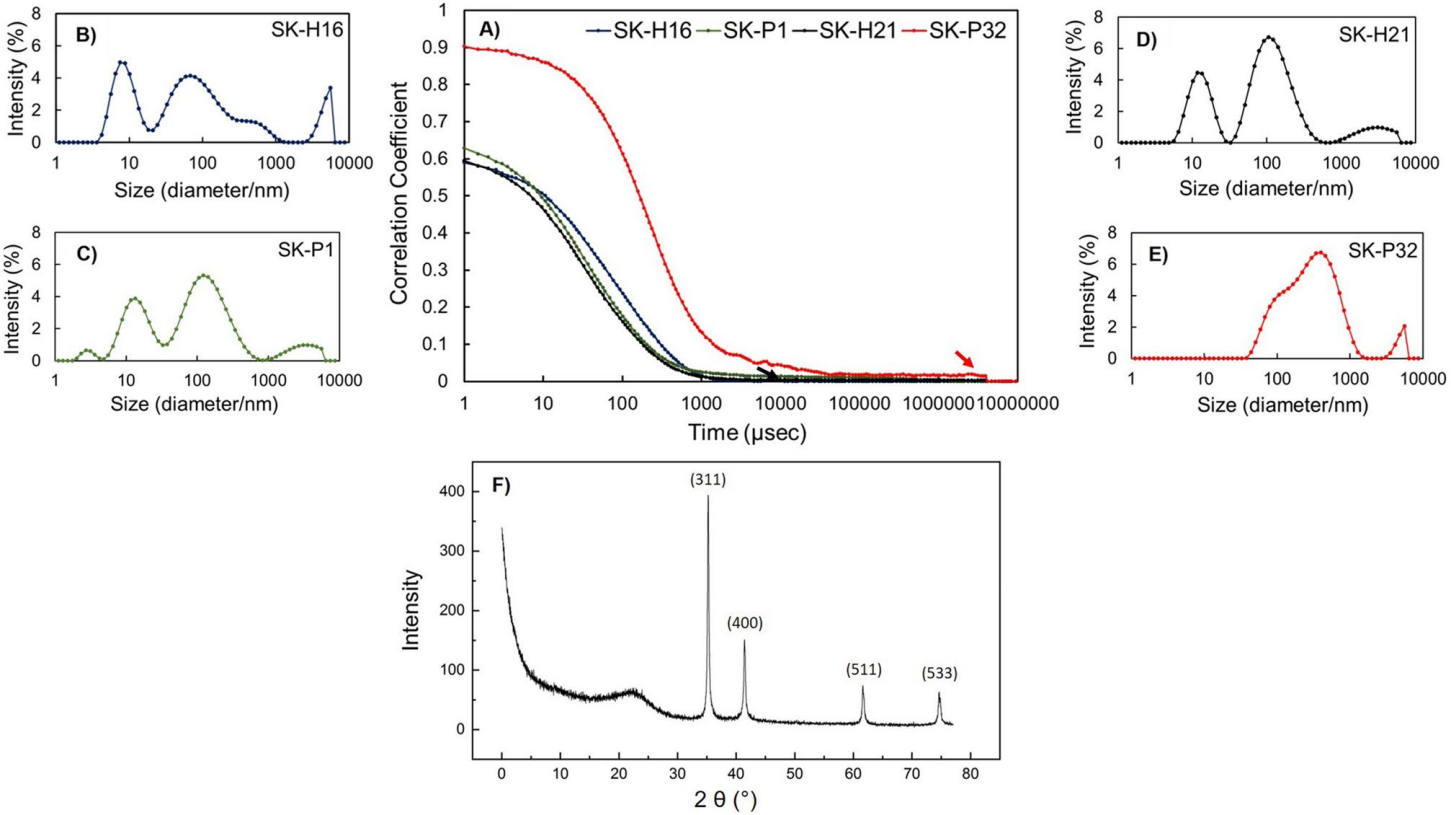
(**A**) DLS analysis of the isolates (SK-H16, SK-P1, SK-H21, SK-P32). (**A**) Three isolates showed a relatively smoother signal decay (black arrow) than the 4th (red arrow)) counterpart; signal decay took longer for this sample as it contained larger sized particles. Intensity distribution graphs (**B**–**E**) indicated that 3/4 isolates (**B**–**D**) had a significant particle population ≤ 100 nm while sample (**E**) showed larger diameters. (**F**) XRD analysis of magnetosomes isolated from the bacterial samples. The well-known (reported) peaks appeared and demonstrated the presence of magnetite.

As SEM provided clear pictures of the morphology and size of the bacterial magnetosomes, we applied DLS to determine the hydrodynamic diameter of the nanoparticles. The correlogram (Fig. 4A) for these measurements highlighted that the signal decay was rather smooth for three samples (H16, H21 and P1) and diminished relatively faster than P32, (black arrow). However, in case of P32 it kept on persisting for a relatively longer time before getting decayed (red arrow). In the intensity measurement analysis, it was clearly seen that the three samples, H16, H21 and P1, all had more one than peaks but the PDI for all of these measurements, however, stayed well below 0.7 and the majority of the particles roughly exhibited a diameter of 40 nm represented by the middle peaks. (Fig. 4B–D). However, the hydrodynamic diameter recorded for P32 was over 200 nm and the presence of a rather broad peak (Fig. 4E) was noticed. This finding further supported the SEM and STEM imaging analysis where the SKP32 sample was found to contain more debris (or severe aggregation) inside it.

#### X-ray diffraction

XRD was performed to confirm that the biosynthesis of magnetic nanoparticles generated magnetite rather than greigite nanoparticles. The XRD results were determined from a mixture of the four isolates to generate a better detection signal with lower noise. Therefore, all four samples were combined, and dried into powder form for this analysis. As seen in the XRD pattern, *P. aeruginosa* cultures had resulted in magnetite nanoparticle formation with a pattern showing clearly (well-known and reported) peaks corresponding to the documented magnetite peaks (at 311, 400, 511, and 533; Fig. 4F). These peaks were in complete agreement with previous reports of XRD of magnetosomes retrieved from aerobic MTB^4^ as well as the ICSD (Inorganic Crystal Structure Database) reference code 01–076-1849.

### Magnetic analysis of bacterial nanoparticles

**Figure 5:**
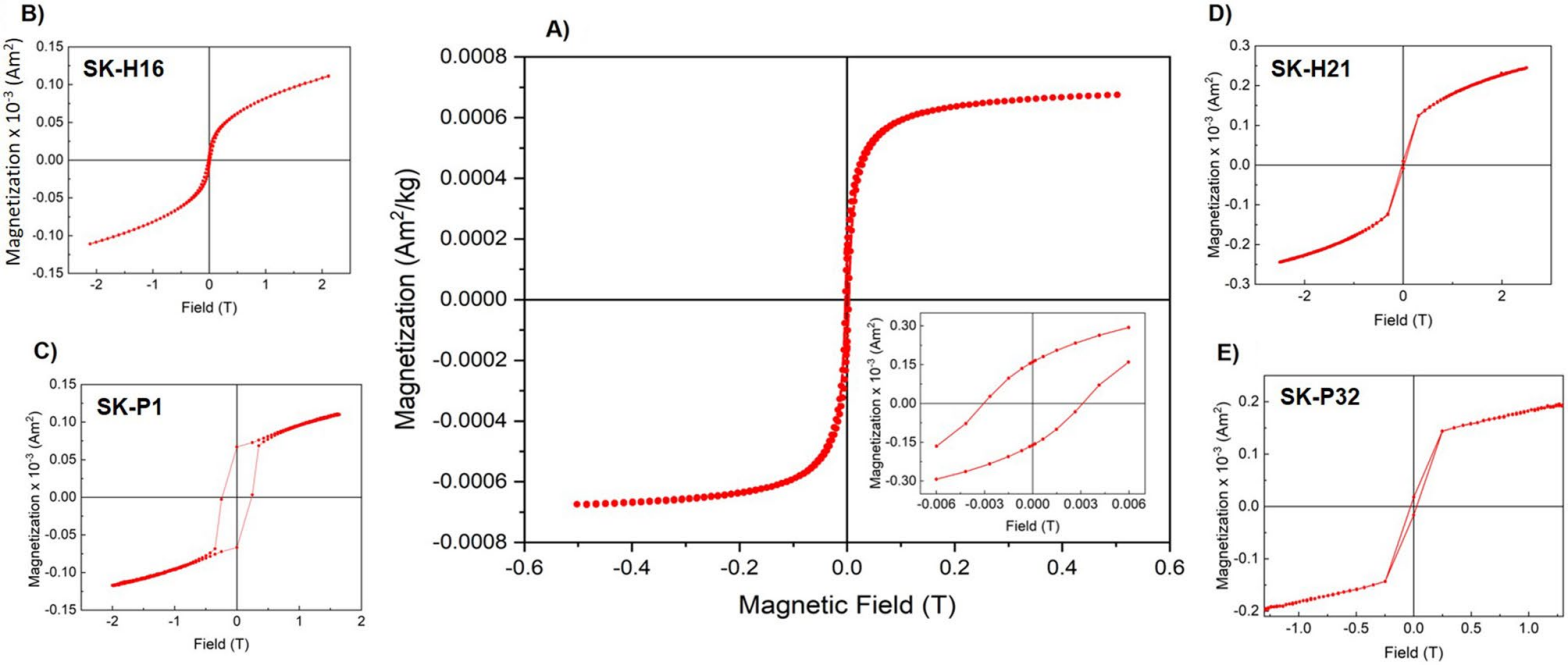
(**A**) Magnetization curve of the magnetosomes showed a superparamagnetic (like) behaviour at 300 K. Inset shows that the particles had a small remanence and coercive fields. (**B**–**E**) Magnetic analysis performed with whole bacterial cells. The hysteresis loops obtained from all of the four *Pseudomonas* strains showed they had magnetic nanoparticles synthesized inside them.

We performed magnetic characterization of the isolated bacterial magnetite nanoparticles after obtaining interesting results from SEM, DLS, and XRD. We detected magnetic behaviour of all batches of the bacterial cells and therefore we performed magnetic analysis for all of them (Fig. 5B–E). However, in these magnetic characterization evaluation we used the whole bacterial cells. We found interesting hysteresis loops where all samples were (nearly) superparamagnetic at room. We saw during these measurements that it was not easy to obtain higher signals from individual particles from each isolate. It was also observed that the hysteresis loops of all the 4 samples were relatively similar and, therefore, we mixed all magnetosomes obtained from the four bacterial strains and used them collectively for magnetic analysis. The magnetization plot on a vibrating sample magnetometer indicated the presence of superparamagnetic like features at room temperature. The highest magnetization moment was 6 × 10^−4^ Am^2^/kg, saturated at 0.2 T against the applied magnetic field (Fig. 5A). It was also evident that small coercive fields (at 0.002–0.003 T) and remanent magnetization were observed upon reversal of magnetic fields (inset: Fig. 5A) and completion of the hysteresis loop. This finding implied that the isolated magnetic nanoparticles were (either) superparamagnetic or ferromagnetic with lower coercivities and remanence.

### Molecular characterization

#### Polymerase chain reaction

**Figure 6:**
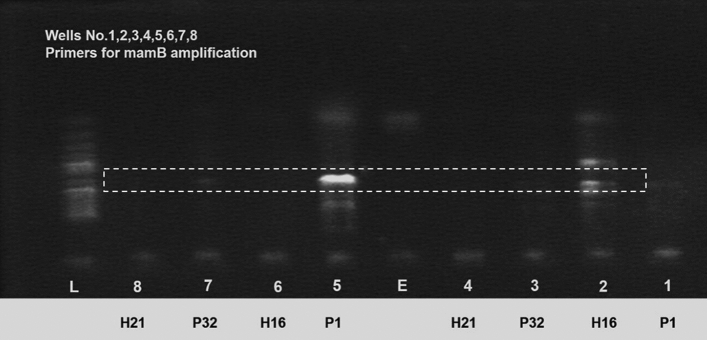
PCR band for *mamB*. P1 and H16 showed strong amplified regions, while H32 had a weak band appearing in the gel after *mamB*-mediated PCR amplification. H21 showed no amplified regions.

PCR amplification of the genome resulted in an amplicon of 534 bps, suggesting the presence of the *mamB* gene. Figure 6 illustrates the presence of *mamB* in three of the four strains of *P. aeruginosa*. The H21 strain could not be amplified using our *mamB* primers, and hence no bands could be seen in the agarose gel. *Mam B*, a cation diffusion facilitator family member, regulates magnetosome-directed iron transport^17^. Deletion mutagenesis studies revealed that *mam B* is essential for forming magnetosome membrane vesicles^18^. The presence of the *mamB* gene suggests the magnetic characteristics of *P. aeruginosa*. The band for P1 is stronger (and appear brighter) than other counterparts and the reason for this is that it has a higher concentration of DNA. The PCR amplification conditions for (at least) this gene were optimum and the efficient amplification resulted in higher quantities of DNA which appeared thicker, stronger and brighter under the UV transilluminator.

## Discussion

The number of culturable MTB is increasing rapidly. According to a report in 2009, 11 were isolated and cultured successfully and it got doubled in 2012 and reached more than 30 till date^19^. Initially, the study of MTB was confined to anaerobic and microaerophilic conditions, however, after the isolation of aerobic MTB, the emphasis had shifted towards the isolates from oxygen containing environments too. The aerobic environment is easy to maintain in laboratory conditions than anaerobic or microaerophilic^20^. The objective of the present study was to biosynthesize magnetic nanoparticles under aerobic conditions that would be superior to microaerophilic MTB, where slightly higher concentrations of oxygen hamper growth conditions. Accordingly, we selected *P. aeruginosa*, an aerobic γ-proteobacterium. It utilizes many pathways and siderophores, facilitating the utilization of iron sources for energy and iron uptake. *P. aeruginosa* has a vast affinity for iron through various intake systems including the haem-source utilization through the ‘Phu system’^13^. For the non-haem sources, *P. aeruginosa* produces chelating siderophores that have high affinity for iron such as pyoverdine and pyochelin^15^. *P. aeruginosa* also have the FeOABC system for iron uptake available to it in the Fe^2+^ form.

Four samples (SK-P1, SK-P32, SK-H16, and SK-H21) were cultured in LB and 9 K medium. Growth rates in 9 K medium for all strains were almost three times less than growth rates in LB. A possible reason for this difference could be that the optimal pH for *P. aeruginosa* in LB is 5.5–6.5. By contrast, in 9 K, pH should be below 3.5, causing the iron ions to precipitate, becoming unavailable for bacterial use. This was shown by Silverman and Lundgren in *Ferrobacillus ferrooxidans*^21^. LB has an organic carbon source available for bacteria in abundance, while 9 K medium possesses minimal carbon sources in dissolved carbon dioxide. This condition, however, ensures the presence of iron ions utilized for energy and magnetic nanoparticle synthesis. Nevertheless, growth on 9 K media was adequate for testing for magnetotaxis and showed positive results when treated with a permanent magnet (see Fig. 2B). The cell culture supernatant and cell lysate could not result in the biosynthesis of the magnetic nanoparticles. There are reports which mention that nanoparticles could also be synthesized on the cell surfaces because of the presence of certain proteins/enzymes^22^. In our case, neither the supernatants nor the (dead) cell-lysate could result in the synthesis of magnetic nanoparticles (Fig. 2C–D) which proved that this process was taking place inside the cells^23^. All bacterial stains slowly aligned to the magnetic field gradient, indicating magnetic particles inside, likely to be magnetosomes. To confirm this, we extracted intracellular magnetosomes biomineralized by *P. aeruginosa* using sodium dodecyl sulfate followed by ultrasonication. The magnetosomes were isolated with the help of a permanent magnet, and the organic/inorganic debris was washed away with saline, followed by washing with distilled water^24^. The analysis of the putative functions of *mam* genes is also important in the interpretation of the evolution of magnetotaxis^25^. Various magnetotactic bacteria possess cluster of mam gene including : mamA, B, C, D, E, F, H, I, K, L, M, N, O, P, Q, R, S, X and Z, in addition to the mms6 and mmsF genes having vital functions in magnetosome synthesis and alignments^26^. Further microbiological characterization of four strains of *P. aeruginosa* confirmed the presence of magnetosome genes. Three (P1, P32 a,d H16) of the four isolates had got amplified in a PCR with *mamB* primers elucidating a horizontal gene transfer event in which they would get these genes from other bacteria. The *mamB* gene is conserved in all species of MTB and plays a crucial role in magnetosome formation^27^.

The purified magnetosomes from *P. aeruginosa* were characterized by several techniques. Electron microscopic studies of these particles revealed much information about their size and morphology. Regardless of composition, magnetosomes displayed a (narrow) size range of 35–50 nm (see particle distribution in Fig. 3); they are reported to appeared in various morphologies (cubo-octahedra, parallelepipedal, or bullet-shaped)^28^; we found it hard to classify our magnetosome in terms of their shapes. The morphology of nanoparticles appeared vague in the SEM images because of the presence of cell debris in the samples and lower resolution. It is logical to encounter such troubles in SEM due to higher amount of organic cell lysate and the (lysis) salt (solutions) which are not always possible to be removed completely. However, it was clear enough in the STEM imaging and the size ranged from 35 to 50 nm in diameter for three samples (H16, H21 and P1); the appearance of magnetosome chains on electron microscopy agreed with Lins et al.^29^. The magnetosomes were clearly showing a no aggregation pattern as they are always found in chains with magnetic nanoparticles (magnetite or greigite) encapsulated in protein sheaths (layers)^30,31^. The protein sheaths acted as a naturally obtained surface stabilizing agents. This would be the reason for good monodisperse samples for magnetic nanoparticles obtained from MTB. However, to determine the exact shape/morphology of our nanoparticles, we will need to develop and optimize better purification assays and use high-resolution TEM imaging in the future. The hydrodynamic diameter measured by DLS was (slightly) greater than the diameter obtained through electron microscopy, explained by the fact that the thin electric dipole layer of solvent adhered in the former case while the latter would give an estimation of the projected area^32^. It is well documented that magnetotactic bacteria biosynthesize magnetite when cultured in the presence of oxygen. Our cultures were grown aerobically with a substantial amount of sulfur salts in the growth medium, therefore it was compelling to determine whether the magnetic nanoparticles would be magnetite, greigite, or amorphous magnetic nanoparticles. Since the natural synthesis here is in an aqueous environment (of the cytoplasm) under mild temperature conditions, amorphous nanoparticles could be produced^33^. XRD peaks at 311, 400, 511, and 533 revealed the presence of magnetic nanoparticles which is consistent with data reported by Talib et al.^4^ and Fischer et al.^4,34^ and corresponded to the ICSD reference code 01–076-1849. The diffraction pattern proves that *P. aeruginosa* under the conditions exploited in this work would synthesize magnetite. At the position of 2θ ranging from (about) 42–58° showed a flat line which pointed to the non-crystal (amorphous) region in the magnetite crystals. However, we noticed the emergence of no new peaks in XRD pattern in the current culture conditions. A critical analytical view would also speculate if the biosynthesized nanoparticles are magnetite or maghemite since they both share very similar XRD patterns and it is not easy to distinguish between them. It is recommended to perform sophisticated characterization methods such X-ray absorption or Electron Energy Loss (EEL) spectroscopies. However, in our case we can rule out maghemite synthesis with high probability as so far no reports for maghemite production inside the magnetotactic bacteria have been published. This makes it easier to distinguish on the basis of XRD pattern if it would be a greigite or magnetite. The magnetic analysis of the magnetic nanoparticles revealed that they were superparamagnetic like as the coercive fields were very low (0.02–0.03 T). We had not deducted the diamagnetic background from the samples. It was not easy to obtain a complete pure magnetite nanoparticles population because of working with very low culture volumes. We believe that upon devising an optimized protocol to increase the nanoparticles yield, better sample purification and deduction of the organic debris, the magnetic moment will be significantly enhanced. The bacterial cells were also evaluated for their magnetic properties and we found that all four of them were magnetic. However, we could not really quantify the masses of the magnetic nanoparticles inside; we, therefore, represent it as the magnetization. It double proved that the bacterial cells had magnetic nanoparticles synthesized inside them. For all these characterizations, we are safe to conclude that *P. aeruginosa* can synthesize magnetic nanoparticles aerobically, they are crystalline magnetite and magnetic.

**Figure 7:**
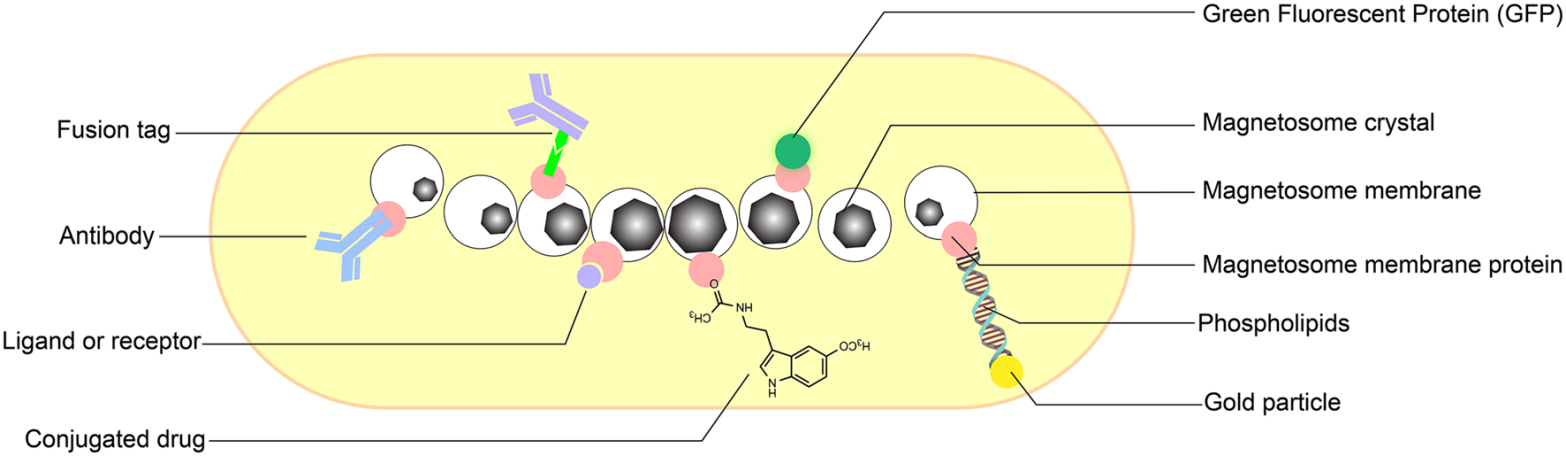
A graphical depiction of the potential applications for the magnetosomes (magnetic nanoparticles) obtained from *P. aeruginosa*.

MTB (*P. aeruginosa*) had been previously isolated from environmental samples. However, the isolation of biomineralizing magnetosomes by bacteria present in clinical samples as reported here is novel and has not yet been reported^24^. *P. aeruginosa* is easily grown under laboratory conditions. Accordingly, scaling up the synthesis of magnetic nanoparticles can be achieved using these strains to replace magnetic nanoparticles' physical and chemical synthesis while enhancing biocompatibility for in vivo and ex vivo applications. Our clinical samples also displayed some disadvantages for using them at pilot scale to synthesize magnetic nanoparticles. These include the presence of pathogenic gene islands and proteins. Nevertheless, they also have potential advantages for recognizing human cell receptors for attachment in the lungs. However, mutation of strains from clinical samples employing deletion of pathogenic genes (without disrupting biomineralizing capability) holds substantial promise for use in vivo. For biological and biomedical applications, magnetic iron oxide NPs are the best choice, for their biocompatibility, superparamagnetic actions, and chemical stability^35^. Magnetic iron oxide NPs have been considered as the best choice, and the application of small iron oxide NPs in in vitro diagnostics has been practiced for almost half a century. MNPs can bind to drugs, proteins, enzymes, antibodies, or nucleotides, and can be absorbed to an organ, tissue, or tumor using an external magnetic field. Furthermore, they can be heated in alternating magnetic fields for use in hyperthermia^36^. Figure 7 depicts many potential applications for our magnetic nanoparticles. The method of biosynthesis through *Pseudomonas* has the advantage of magnetic nanoparticles of a narrow-size distribution making potential candidates for magnetic hyperthermia. MNPs with narrow-size distribution helps in maintaining temperature needed according to the exact calculations for cancer treatment.

## Materials and methods

### P. aeruginosa isolates/samples

A total number of four clinically isolated *P. aeruginosa* samples were obtained from the Microbiology & Public Health Lab, COMSATS University Islamabad, as glycerol stocks. They were named SKP1, SKP32, SKH16, and SKH21.

### Preculture of the isolates

The *P. aeruginosa* samples were cultured in Luria Bertani (LB) broth and incubated on a shaking incubator at 30 °C. The thriving bacterial cells were confirmed to be *P. aeruginosa* using several biochemical fingerprinting tests.

### Culture in the chemical medium

The isolates were cultured in a simple 9 K medium to isolate magnetotactic bacteria as previously reported^4,37^. The microorganisms were incubated at 37 °C on a shaker at 120 RPM. They were monitored by measuring optical density (OD) after every 3 h to determine their pattern via making a growth plot.

### Biochemical characterization

After the isolates were re-cultured and found to be pure, we reconfirmed them using biochemical methods. The bacterial cells were Gram-stained and visualized under a light microscope. They were also cultured on a selective cetrimide agar under aerobic conditions for 7 days to observe the growth and pigment appearance. Other biochemical tests included oxidase, catalase, indole, H_2_S, and motility tests, and Simmon’s citrate agar culture for *P. aeruginosa* reconfirmation.

### Magnetic movement

Bacteria were cultured in 9 K medium to determine the presence of bacteria exhibiting magnetotactic properties. Therefore, 1 mL of bacteria was harvested at the log phase. They were spun at 12,000 g for 5 min, the pellets were washed three times with 0.9 M NaCl, and the supernatants were discarded. The bacterial pellets were resuspended in dH_2_O, and a strong permanent magnet (0.2 T) was placed at one side of the tube harboring the cells. To confirm that the magnetic nanoparticles were synthesized by the cells inside the cytoplasm and not either on the cell surfaces or release of enzymes into the medium, we performed two experiments. Firstly, the P. aeruginosa cells were cultured in LB medium until 0.8–1.0 OD (600 nm). The cells were spun at 3000 g and supernatant was mixed with 9 K medium and incubated. Secondly, the cultured cells were raptured and the LB containing debris was mixed with 9 K medium. In both of the aforementioned cases, the incubation was kept similar to what was used for magnetosome biosynthesis.

### Extraction of magnetosomes

Confirmed MTB strains were cultured in 200 mL 9 K medium in conical flasks with shaking. A volume of 50 mL was harvested at the log phase and centrifuged at 13,000 g for 10 min at 4 °C. The pellets were washed three times with 0.9 M NaCl followed by suspension in 0.2% sodium dodecyl sulfate and incubation at room temperature for 45 min. The suspended bacteria were sonicated (Q Sonica, Probe Sonicator) at 20 kHz for 10 min at 40% amplitude (pulsed with pulse off and on for 1 and 2 s, respectively). Magnetosomes were collected after sonication by applying a strong magnetic field through a permanent magnetic bar at one side of each tube. The extracted magnetosomes were added to Eppendorf tubes and washed in 0.9 M NaCl. The final product was stored in phosphate-buffered saline at 4 °C.

### Electron microscopy

Scanning Transmission Electron Microscopy (STEM) was performed in a 400-mesh Cu grid coated with film of carbon (Agar Scientific). The surface of the grid was treated to make it more hydrophilic and a sample volume of 5–7 µL was poured on it. The sample was let to deposit on the grid for 10 min, the excess fluid was removed through a (non-fibrous) tissue paper and was installed in the STEM (Helios NanoLab™, FEI, Netherlands) for imaging.

Magnetosomes obtained at high concentrations were used for X-ray powder diffraction (XRD) analysis. Magnetosomes isolated in pure form were dried on clean glass slides. The samples were reduced in size as much as possible and were added to the sample holder. An X-ray diffractometer (X’Pert, PANalytic, Netherlands) in reflection mode was used for phase identification.

### Dynamic light scattering

Dynamic light scattering (DLS) was performed to obtain a clear picture of the size (hydrodynamic diameter) of the magnetosomes/magnetic nanoparticles. We employed standard methods, as described^4,38^.

DNA extraction and polymerase chain reaction amplification of the mam B gene.

DNA was extracted from the bacterial cells through the boiling method^39^. The polymerase chain reaction (PCR) was performed to amplify the *mam B* gene in the *P. aeruginosa* genome. The primers used {R-(5′ TACCGCCTCGGCCACCAT 3′) and F-(5′ ATGAAGTTCGAAAATTGCAGRGA 3′)} would result in an expected amplicon of 548 bps^10^. A standard PCR protocol for 30 cycles was followed. The PCR products were run on agarose gels for analysis.
